# Fluorogenic RNA-Based Biosensors of Small Molecules: Current Developments, Uses, and Perspectives

**DOI:** 10.3390/bios14080376

**Published:** 2024-08-01

**Authors:** Janine Kehrli, Claire Husser, Michael Ryckelynck

**Affiliations:** Université de Strasbourg, CNRS, Architecture et Réactivité de l’ARN, UPR 9002, F-67000 Strasbourg, France; janine.kehrli@etu.unistra.fr (J.K.); claire.husser@etu.unistra.fr (C.H.)

**Keywords:** fluorogenic RNA-based biosensors, fluorescent reporters, RNA, synthetic nucleic acids, small molecule, light-up aptamer, selection, structure-switching aptamer, FLAP, molecular imaging

## Abstract

Small molecules are highly relevant targets for detection and quantification. They are also used to diagnose and monitor the progression of disease and infectious processes and track the presence of contaminants. Fluorogenic RNA-based biosensors (FRBs) represent an appealing solution to the problem of detecting these targets. They combine the portability of molecular systems with the sensitivity and multiplexing capacity of fluorescence, as well as the exquisite ligand selectivity of RNA aptamers. In this review, we first present the different sensing and reporting aptamer modules currently available to design an FRB, together with the main methodologies used to discover modules with new specificities. We next introduce and discuss how both modules can be functionally connected prior to exploring the main applications for which FRB have been used. Finally, we conclude by discussing how using alternative nucleotide chemistries may improve FRB properties and further widen their application scope.

## 1. Introduction

Small molecules are defined as compounds having molecular weights lower than 1000 daltons. Although they only partially fit this definition, ions will also be considered herein, as they share functional importance and properties with small molecules. This very large and diverse class of compounds can be of natural (biological or abiological) or synthetic origin and comprises families of molecules as diverse as vitamins, metabolites, neurotransmitters, hormones, sugars, antibiotics, toxins, synthetic drugs, pesticides, and industrial wastes, just as a few examples. Being able to detect and quantify these small molecules paves the way to a tremendous number of applications in diagnostics, infectiology, food safety control, and in the tracking of environmental pollutants [1,2]. Conventional analytical chemistry usually tackles this problem using chromatographic and mass spectroscopic characterizations, often performed in tandem [3].

**Figure 1:**
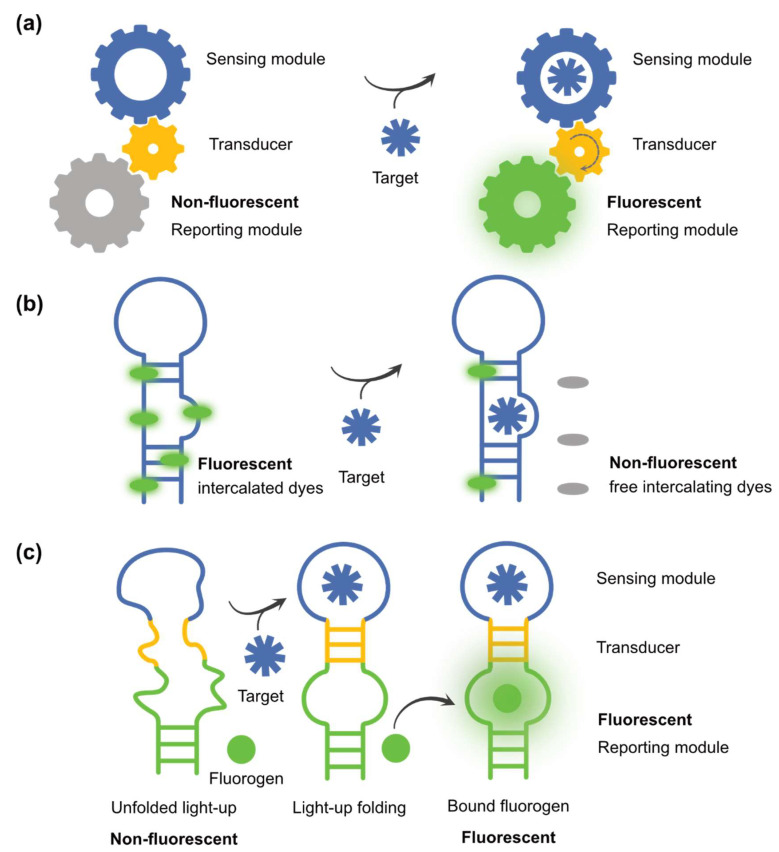
Principle of fluorogenic biosensors for small molecules. (**a**) General working principle of a fluorogenic biosensor. In absence of the ligand, the biosensor is in a dark conformation preventing fluorescence emission. In the presence of the ligand, the biosensor undergoes a structural rearrangement leading to fluorescence emission. (**b**) Working principle of a biosensor exploiting intercalating dye displacement. The sensing nucleic acid is turned fluorescent upon staining by a nonspecific fluorogenic intercalating dye. The recognition of the target small molecule leads to dye displacement and concomitant fluorescence reduction. (**c**) Nucleic-acid-based fluorogenic biosensor. The molecule is made of a light-up aptamer (green) functionally connected to a sensing aptamer (blue). In absence of target ligand, the structure of the light-up aptamer is destabilized, and its fluorescence capacity is abrogated. However, the binding of the target ligand to the sensing aptamer, induces a structure switching that the transducer module (yellow) transmits to the light-up aptamer. The latter is then stabilized; it recovers its capacity to bind its fluorogen and forms a fluorescent complex. Arrows symbolize molecular recognition events between RNA and target ligand or specific fluorogen.

Despite their high sensitivities and applicability to a wide range of targets, these technologies require invasive sample preparation; they are expensive and use pieces of equipment that are difficult to move. This lack of portability makes them poorly compatible with on-site and point-of-care applications. Yet, several of these limitations can be overcome by biosensing. A biosensor is a device that is made of at least two components, a sensor and a reporter, that should be functionally connected. The biosensor includes at least one biological component (e.g., enzyme, antibody, or nucleic acid) that reports on the presence of a target molecule by the emission of a measurable signal (Figure 1a) [4]. In some cases, a biosensor is a whole reprogrammed cell, but this situation will not be further discussed in this article and the reader is redirected to a recent review on this topic [5].

The first biosensor was introduced in 1962 by Clark and Lyons to sense glucose using an enzyme (i.e., glucose oxidase) functionally linked to a pH-sensitive electrode by the proton exchange resulting from the reaction [6]. This first prototype later led to the development of a plethora of electrochemical biosensors [7]. With ever-improving electronics and fabrication techniques, such biosensors have now reached a great degree of miniaturization [8]. However, as they rely on the enzymatic conversion of the target analyte, their use is restricted to small molecules for which a catalyst exists. This approach can, nevertheless, be adapted to an ELISA-like format if a pair of target-specific and -compatible antibodies (one immobilized antibody displayed at the surface of the sensor to capture the analyte and a second coupled to a reporting enzyme) is available, which remains a significant bottleneck.

Besides electrochemistry, fluorescence is likely the other most-used reporting strategy, as it offers several advantages. First, the use of proper filter sets and state-of-the-art detectors enables the setup of highly sensitive assays. Second, as fluorescent reporters are excited and emit fluorescence in well-defined spectral windows, multiplexed assays can easily be devised. Finally, noninvasive fluorescent assays can be set up and data collected directly from living cells while preserving their integrity using genetically encoded biosensors directly synthesized by the cell itself. The discoveries of the green fluorescent protein (GFP) [9] and its derivatives enabled the development of many protein-based fluorescent biosensors using a fluorescent protein (FP) as a reporting module [10,11]. The sensing module can be taken from the vast collection of proteins (e.g., transcription factors, ligand receptors, and transporters) known to be able to specifically interact with the target molecule [12,13]. This has enabled the creation of various fusion proteins emitting fluorescence in response to small-molecule detection. Comprehensive reviews on this topic can be found elsewhere [14,15,16,17]. Yet their development is often long and tedious, and the isolation of a new sensing domain (e.g., by reprogramming the specificity of an existing one) remains a rather complex task.

Nucleic acids represent an attractive alternative to proteins. The capacities of ribonucleic acids (RNAs) to specifically interact with small molecules have been known for a long time [18]. On the one hand, many organisms evolved to produce antibiotics specifically targeting bacterial ribosomal RNA [19]. On the other hand, bacteria also acquired RNA motifs, called riboswitches, able to specifically sense small molecules and adapt gene expressions accordingly. Besides these natural motifs, synthetic RNA-sensing modules can also be identified de novo, improved, or even reprogrammed using in vitro selection and evolution technologies that will be depicted in more detail below. Upon obtention, these sensing modules can be converted to biosensors using reporting strategies exploiting electrochemistry [20], nanoparticle-selective aggregation [21], or fluorescently labelled oligonucleotides [22,23,24]. Whereas these technologies are well suited for in vitro target detection, they are more limited for applications in living cells and organisms. This challenge can be partially overcome using a genetically encodable RNA-based sensor. However, in contrast to proteins, no naturally fluorescent RNA has been discovered so far, leading to significant efforts in developing approaches aimed at converting native ligand-specific structure-switching nucleic acids to fluorescent reporters. Such sensors can first be obtained by staining a sensing RNA module with a fluorogenic intercalating dye to turn it fluorescent [25]. The binding of the target small molecule then induces dye displacement, quenching, or sometimes recruitment, followed by variations in absorbance or fluorescence (Figure 1b). Although functional and suited for in vitro applications, the use of such assays in living cells is strongly challenged by the lack of specificity of the fluorogen and the elevated cellular contents in competitive nucleic acids. This limitation can, however, be overcome using light-up RNA aptamers, i.e., small single-stranded nucleic acids adopting a three-dimensional structure, allowing them to specifically interact with and activate the emission capacity of a fluorogen. These light-up aptamers can be converted into target-specific biosensors by coupling them with a target-sensing aptamer (Figure 1c). In this short review, we will first present how both reporting and sensing aptamer modules can be isolated, prior to introducing the main strategies to functionally associate them into efficient fluorogenic RNA-based biosensors (FRBs). After having explored some applications made possible by these FRBs, we will conclude by discussing how using alternative chemistries may further expand the future application range of these biosensors.

## 2. Reporting and Sensing Modules

As stated above, an FRB is typically made of a target sensor functionally connected to a reporting module. Although both modules are expected to specifically bind a small molecule (the target or the fluorogen), the way they are obtained may significantly differ.

### 2.1. Light-Up Aptamers Discovery and Properties

A wide variety of light-up aptamer/fluorogen pairs has been developed over the past decade with colors spanning the entire visible spectrum (Table 1), some systems displaying great color flexibility [26,27,28]. Their deep description and characterization can be found in several recent excellent reviews on the topic [29,30,31,32,33,34].

**Figure 2:**
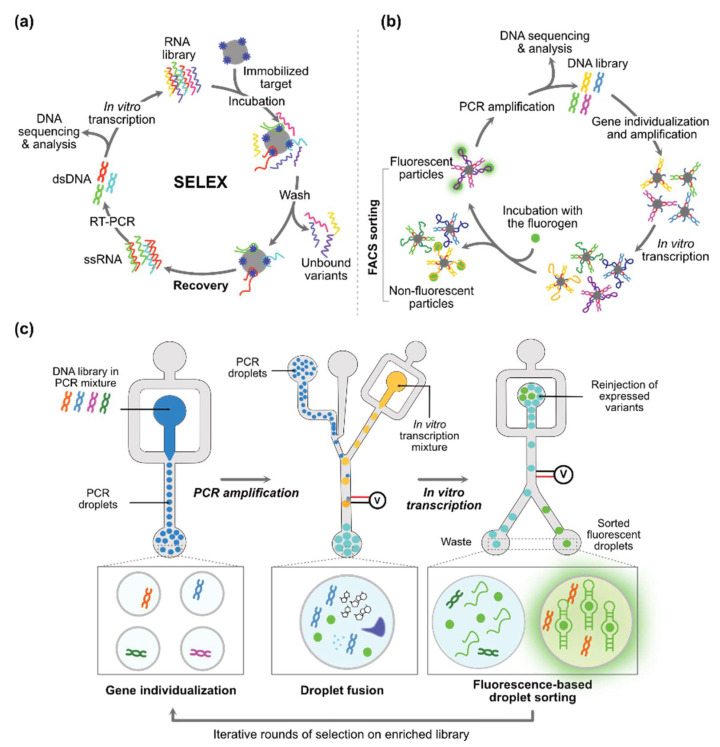
Main approaches to isolate light-up aptamers. (**a**) Systematic evolution of ligands by exponential enrichment (SELEX). During this in vitro selection process, an oligonucleotide (DNA/RNA/XNA) library is generated and incubated with beads coated with the aptamer ligand. Aptamers that remain attached to the beads after multiple washing steps are recovered. Each round of selection allows the gradual enrichment of aptamer based on their affinity for the target. (**b**) Fluorescence-activated cell sorting (FACS)-based selection of aptamers displayed on beads and particles. DNA genes contained in the library are individualized and amplified, at the surface of the particles, followed by in vitro transcription and incubation with the fluorogen. FACS is used to sort particles coated with the aptamer of interest (i.e., the most fluorescent particles). Aptamers are therefore directly selected for their ability to activate the fluorogen fluorescence. (**c**) Microfluidic-assisted in vitro compartmentalization. The starting DNA library is encapsulated into water-in-oil droplets (2.5 pL) using a droplet generator microfluidic chip. The emulsion is collected, and DNA contained in each droplet is amplified by PCR. Droplets are then fused with a droplet containing all necessary reagents for in vitro transcription as well as the fluorogen (green dot). Droplets are finally reinjected into a sorting device, where fluorescence emission is measured, and the droplets are sorted accordingly.

Most of these synthetic aptamers were originally isolated using systematic evolution of ligands by exponential enrichment (SELEX) [68,69]. This in vitro selection approach consists of isolating nucleic acids contained in a large library (up to 10^16^ variants) for their ability to specifically bind their fluorogen immobilized on a solid phase (Figure 2a). These libraries are frequently made of a 30-40-nucleotide-long randomized stretch interrupted by a stem-loop structure, as the presence of such an internal prefolded element was found to drastically increase the success rate of SELEX targeting small molecules [70]. Whereas this approach is highly effective for selecting binders, it does not allow any selection pressure to be applied for their capacity to trigger fluorescence emission. Therefore, following the SELEX step, enriched libraries are usually cloned and some constructs are functionally tested either in vitro or upon expression in living cells (mostly in bacteria) later tested in microtiter plates [28,49,60,63]. Upon incubation with the target fluorogen, cells displaying the best fluorescence signal can be analyzed and sorted by FACS [36,38,67]. Alternatively, genes contained in the libraries can be isolated at the surface of beads [70], of microparticles (Figure 2b) [71,72], or within water-in-oil microfluidic droplets (Figure 2c) [47,51,56] where they are amplified and expressed. In each case, fluorogen addition turns the particle (compartment) fluorescent according to the functionality of the encoded aptamer, making it possible to sort the particles of interest by FACS or the droplets using a fluorescence-activated droplet sorter [73]. Finally, the overall selection/screening process can be characterized a posteriori using high-throughput sequencing and variants of interest identified through bioinformatics [74].

**Figure 3:**
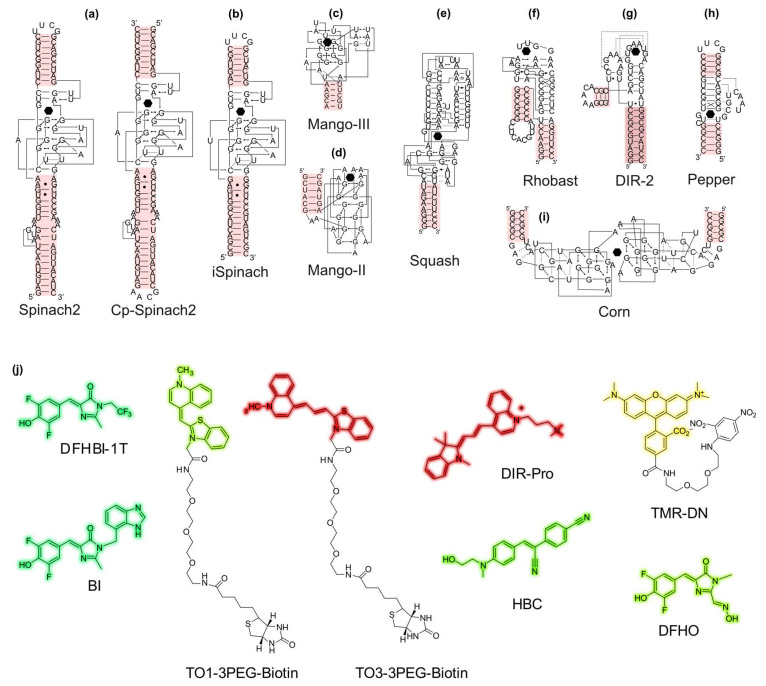
Structural organization of the principal light-up aptamers and their cognate fluorogens. The sequence and two-dimensional structure of the light-up aptamer is represented according to crystal structures and to Leontis–Westhof representation. The fluorogen is represented by a black dot. Possible entry points usable to design a fluorogenic RNA-based biosensor are highlighted in red. (**a**) Spinach2 aptamer [65] and its circular permuted version (Cp-Spinach2) [75], (**b**) iSpinach aptamer [48], (**c**) Mango-III aptamer [53], (**d**) Mango-II aptamer [52], (**e**) Squash aptamer [67], (**f**) RhoBAST aptamer [61], (**g**) DIR2s aptamer [45], (**h**) Pepper aptamer [59], and (**i**) Corn aptamer [43]. (**j**) Chemical structure of the main fluorogens specifically activated by a light-up RNA aptamer. The fluorogenic part of the molecule is shadowed in a color matching its emission wavelength. Aptamer/fluorogen compatible pairs are listed in Table 1.

The crystal structure of most light-up aptamer/fluorogen pairs has now been solved (Table 1), providing extremely valuable information on the recognition mechanism between the aptamer and its cognate fluorogen, but this may also guide the development of an improved version of the fluorogen [39]. The 17 structures currently available (Table 1) highlight some general features shared by all or part of these aptamers. In every case, the fluorogen binding pocket is made by an extended planar platform made of a G-quadruplex (Spinach, Broccoli, Mango, Corn, Beetroot, and Peach), a base quadruple (RhoBAST), or a base triple (DIR-2 and Pepper). Most of the time (except for Mango, Mango-II, and Peach), the fluorogen is accommodated either in a binding pocket or at the interface of two aptamers (Corn and Beetroot) that maintains the fluorogen in a planar conformation while restraining solvent accessibility. The fluorogen-binding pocket either lies at the apex of the structure (Mangos, DIR-2, and RhoBAST) or sits in its middle. Moreover, at least one (Mangos, Corn, and Beetroot) and up to five paired regions (o-Coral) surround this binding site and maintain its structure. Interestingly, some of those stems represent a possible entry point in view of future engineering (Figure 3). To date, 40% of these light-up aptamers have been successfully converted into biosensors of small molecules (see Section 4).

### 2.2. Choice and Development of Sensing Aptamers

The sensing module is the second key module of an FRB. Whereas, in rare cases, introducing simple mismatches in a helix is sufficient to sense a metal ion (e.g., C–C mismatch signals the presence of silver by the formation of a C-Ag-C metallobase pair) [76,77], the specific detection of a small molecule usually requires a whole module. Moreover, to yield an efficient biosensor, target-binding should trigger a structural change in the sensing aptamer that will be transmitted to a functionally connected light-up aptamer module. A natural target-specific aptamer can be either directly used or reprogrammed toward a new specificity. Finally, structure-switching aptamers can be developed de novo using dedicated methods.

#### 2.2.1. Repurposing Natural Structure-Switching Aptamers

Structure-switching aptamers are naturally found embedded within riboswitches, a class of *cis*-regulatory RNA mostly found in the 5′ untranslated region of bacterial genes and operons and that control the expression of downstream genes at a transcriptional and/or at translational level (see [78] for a recent comprehensive review on the topic). Aptamers of natural origin arise in intracellular mediums with particular physicochemical conditions, a crowding induced by the presence of numerous macromolecules and a large number of competing small molecules. Consequently, these aptamers are well adapted to work in cellular environments, with good folding efficiencies, high specificities, and physiologically relevant affinities for their target.

The crystal structure of many of these aptamers has been solved, helping to guide their engineering. Yet, of the over 55 classes of riboswitch currently known (responding to ions, vitamins, purines, and nucleotide-derived cofactors), only ~20% entered in the design of an FRB, among which those responding to cyclic dinucleotides and S-adenosyl-methionine (SAM) have been particularly used. Therefore, riboswitches represent a rich reservoir of aptamers for developing new FRBs, especially when considering that the ligands of ~100 orphan riboswitches remain to be identified [78,79,80]. Despite this impressive source of natural aptamers, they are limited to the recognition of molecules that can be found in cells, which represents a serious bottleneck and justifies the efforts undertaken to identify synthetic structure-switching aptamers.

#### 2.2.2. Reprogramming the Specificity of Natural Structure-Switching Aptamers

Riboswitches have evolved over a long period of time to acquire their structure-switching properties. Therefore, they are appealing scaffolds for the isolation of structure-switching aptamers with new specificity. This can be achieved by introducing mutations into the ligand-binding region while preserving the overall aptamer structure. In the simplest case, the specificity of an aptamer whose crystal structure is known can be reprogrammed by the rational mutation of residues essential for ligand recognition. One of the best-known examples is the reprogramming of the xpt-pbuX aptamer [81]. Indeed, while the specificity of this aptamer was driven by the formation of a Watson Crick base pair between the target guanine and the C74 nucleobase, this aptamer was reprogrammed toward adenine only by mutating this position to U74 [82]. The in-depth study of the structure of the different classes of riboswitches and their mode of interaction with their target also led to the development of a biosensor based on the GEMM-II riboswitch scaffold capable of recognizing 2′,3′-cGAMP [83]. However, the possibility of performing such rational design remains marginal and deeper reshaping of the ligand-binding site is often required.

Porter and colleagues nicely demonstrated that the three-way junction (3WJ) motif found in several natural RNAs can provide a robust structural scaffold for the search of synthetic structure-switching aptamer [84]. The authors first randomized every unpaired residue of the core junction as well as the first proximal base pair of each stem of two riboswitches (guanine-specific xpt-pbuX aptamer of *Bacillus subtilis* and cyclic di-GMP-specific Vc2 riboswitch of *Vibrio cholerae*) and one ribozyme (*Schistosoma mansoni* hammerhead ribozyme) prior to subjecting the libraries to several rounds of SELEX. Applying moderate selection stringency and using a high specificity reverse transcriptase enabled the identification of structure-switching aptamers specific to 5-hydroxy-L-tryptophan or to 3,4-dihydroxy-L-phenylalanine. Interestingly, although aptamers isolated from each library differ in terms of sequence and framework, they all functionally converge in terms of affinity and specificity, and they preserved the overall structural organization of their parental molecule.

The concept of reprogramming robust riboswitch-derived scaffolds was deeper explored by the Jaffrey lab using the adenine-specific aptamer module from the add riboswitch of *Vibrio vulnificus* [66]. Not only was the ligand-binding pocket (the residues of the junctions as well as the first proximal base pairs of each stem) of this 3WJ-containing aptamer randomized but the use of a “Sprouts & Clips” solid-phase synthesis strategy enabled its size to be varied. Upon several rounds of SELEX and FACS sorting, the authors were able to isolate Squash, a light-up aptamer characterized by very high folding efficiency, likely inherited from the parental scaffold, allowing it to outperform its functional counterparts (i.e., Broccoli and Corn isolated by conventional SELEX using a pre-folded but unscaffolded library).

#### 2.2.3. De Novo Discovery of Synthetic Structure-Switching Aptamers

**Figure 4:**
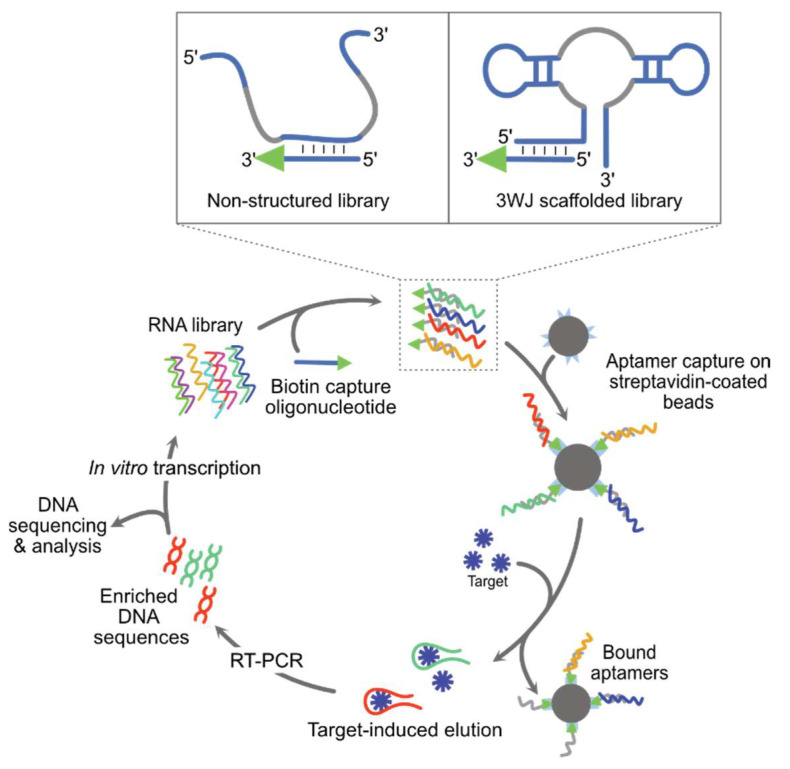
Capture-SELEX. Nucleic acid (DNA/RNA) libraries are incubated with a complementary biotinylated capture-oligonucleotide and incubated with streptavidin-coated beads. The library can be either unstructured (left scheme in the box) or 3WJ-scaffolded (right scheme in the box). The library is immobilized on the beads through the capture-oligonucleotide. Upon extensive washing, the addition of the target molecule induces a structure-switching event that undocks the variants of interest. The latter are then recovered and used for subsequent rounds of selection and/or sequence identification.

Most of the aptamers identified by classical SELEX are not expected to have an efficient structure-switching capacity, despite this being a critical feature for the development of an FRB. However, the isolation of such molecules could be favored using Capture-SELEX. In this approach, the oligonucleotide library is first immobilized on a solid phase (beads) via a capture oligonucleotide (Figure 4) [85]. After stringent washing steps, the aptamers are eluted by addition of the free target. Advantageously, the use of the target in its native form is expected to both simplify the overall process and increase the likelihood of isolating an efficient aptamer. While Capture-SELEX is mainly used to identify DNA aptamers, the Suess group adapted the procedure to RNA chemistry to successfully isolate sensors of rATP [86], paromomycin [87], tobramycin [88], and levofloxacin [89]. Although functional, the conversion of these aptamers into FRBs may, however, be challenged by the internal location of the docking sequence (Figure 4), i.e., where the impact of structure switching is expected to be the highest. This limitation was recently partly overcome by the Mayer lab who introduced a modular approach in which aptamers isolated from Capture-SELEX can be directly coupled with a light-up module, allowing a TPP-specific FRB to be identified [90]. Yet, this biosensor displays a limited response amplitude, suggesting a suboptimal folding.

An appealing strategy for the de novo discovery of efficient structure-switching aptamers exploits a variant of Capture-SELEX in which a 3WJ-scaffolded mutant library is docked at the level of a P1 stem that can directly serve as an entry point for future engineering (Figure 4) [91]. Mutant libraries of the guanine-specific aptamer xpt-pbux from *Bacillus subtilis* allowed the identification of new quinine-, caffeine-, and guanine-specific aptamers functional both in vitro and in vivo, likely thanks to their 3WJ scaffold.

## 3. Functional Connection of the Sensing with the Reporting Modules

**Figure 5:**
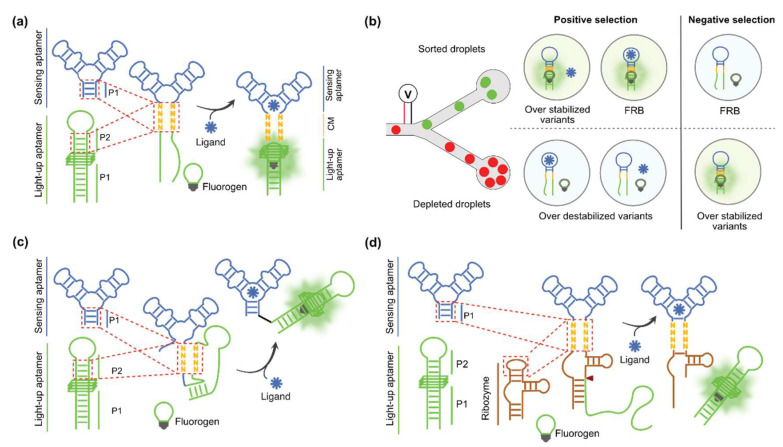
Main design strategies to generate fluorogenic RNA-based biosensors. (**a**) Direct allosteric control: the sensor aptamer (in blue) is directly connected to the light-up aptamer (in green) by merging essential stems through a communication module (CM in orange). The latter transiently destabilizes the light-up aptamer in absence of the ligand but enables its stabilization upon ligand binding, leading to the restoration of fluorogen binding capacity and to fluorescence emission. (**b**) Microfluidic-assisted screening used to select FRBs with optimal communication module. The same microfluidic-assisted pipeline described on Figure 2c is used but iterative rounds of positive and negative selections are performed. During a positive selection, the droplets containing a light-up/fluorogen fluorescence signal in the presence of the ligand (over stabilized variants or optimized FRB) are sorted and the dark droplets (destabilized variants) are discarded. Then, negative selection is performed in absence of ligand and droplets remaining dark (expected FRB) are sorted, while fluorescent droplets (ligand-independent over stabilized variant) are discarded. (**c**) Strand displacement-mediated control. The sensing aptamer (in blue) is connected to the light-up aptamer (in green) replacing the natural riboswitch regulation platform. Transducer sequence (in orange) is designed so that a strand invades and destabilizes the light-up aptamer in absence of ligand. The binding of the ligand triggers the displacement of the invading strand and leads to refolding of the light-up and the fluorogen fluorescence activation. (**d**) Mixed control. The sensing aptamer (in blue) is directly connected to a ribozyme (in brown) through a CM (in orange). One strand of the light-up aptamer (in green) is sequestered by the ribozyme, preventing its folding and abrogating its fluorogenic capacity. Upon recognition of the ligand, the ribozyme self-cleaves and releases the light-up strand that refolds and activates the fluorescence of the fluorogen. Arrows symbolize molecular recognition between the RNA and the target ligand.

Beside the individual properties of the sensing and the reporting modules, the performance of a biosensor also strongly relies on the quality of their functional connection. Indeed, an optimal FRB biosensor of small molecules should exhibit minimal fluorescence in the absence of the target molecule, while emitting the highest possible fluorescence in its presence. Sensing and reporting modules can first be functionally coupled by protein-based molecular machineries [92,93]. For instance, the sensing aptamer can be used in its natural context (i.e., embedded within a riboswitch) to control the transcription of a DNA fragment templating a reporting light-up aptamer [94,95]. This strategy comes with some signal amplification (several light-up aptamers can be produced from a single template). A much higher amplification was achieved in the SPRINT technology [96], which combines the use of riboswitch-mediated transcription control and Cas13a collateral cleaving activity, reported using dually labelled substrate RNA oligonucleotides. To the best of our knowledge, this detection strategy has never been applied to sense small molecules using light-up aptamers as reporters. However, Cas-based methodologies were developed to report on the presence of target RNA [97,98] or even DNA [99] by the synthesis [98,99] or degradation of a light-up aptamer [97]. Despite the substantial gain of sensitivity offered by these protein-mediated approaches, they cannot easily be implemented within living cells and they do not allow fluctuation of small molecule content to be monitored in a dynamic manner throughout an experiment. Nevertheless, both limitations can be overcome using FRB at the cost of a lower sensitivity. Upon target ligand binding to an FRB, the allosteric control event can be exerted in two manners: (i) a direct allosteric control mediated by the stabilization of a dynamic stem or (ii) a structural reshaping through a strand displacement mechanism (Figure 5).

### 3.1. Direct Allosteric Control

A-type helix (also called paired region, or P in short) is the most common, simple, and predictable structural RNA motif, making it an appealing entry point for engineering purposes. Consequently, most FRBs were obtained by identifying a helix on each module to connect (one near the ligand-binding site and the second near the fluorogen-binding site; see possible entry points of light-up aptamers on Figure 3) and merging them in a single stem (Figure 5a). Although candidate helices can be chosen from 2D structural models, the knowledge of the crystal structure of a module greatly facilitates the task. Moreover, further flexibility in the choice of the entry points can be obtained by preparing circularly permuted mutants (i.e., by connecting the 5′ and 3′ ends of the RNA by a tetraloop prior to generating new 5′ and 3′ ends by cleaving a closing loop) [75]. The length (typically one to four base pairs) and sequence of this connecting stem, also known as transducer or communication module (CM), is then adjusted to transiently destabilize the fluorogen-binding pocket (Figure 5a). Therefore, in the ligand-free state, the fluorogen-binding site is expected to be destabilized, which prevents interaction with the fluorogen and keeps the FRB in a dark state. However, the presence of the target ligand induces a structural change in the sensing module that stabilizes the communication module and restores the fluorogen-binding capacity of the reporting module, ultimately leading to fluorescence emission. Overall, this strategy yields FRBs displaying a good dynamic range and a rapid and reversible response enabling dynamic monitoring of target ligand concentration, many of them having been functionally validated in living cells (see Section 4).

Most of the time, the CM is designed in a trial-and-error manner where a few sequences are designed considering thermodynamic criteria prior to being individually tested, with the best-performing one being selected. Nevertheless, the required dynamic behavior remains difficult to predict in silico and these modules are often sub-optimal. As an alternative, a library of randomized communication modules can be prepared and the best candidates are isolated by Capture-SELEX [85] or particle display [72]. Our group also introduced a microfluidic-assisted pipeline in which every variant of the library is individually amplified, expressed, and evaluated in water-in-oil droplets through positive and negative rounds of screening (Figure 5b). Subsequently, the whole process is monitored by high-throughput sequencing and bioinformatics [100]. Such deep analyses of large sequence sets enable the rapid isolation of optimal communication modules, even when only a very low number of solutions exist in the library [101].

### 3.2. Strand Displacement-Mediated Control

FRBs can also be obtained by replacing the regulation platform of a natural riboswitch by a light-up aptamer module [102,103]. Then, sequence variations are introduced into the sensing and/or the light-up modules to generate a transducer sequence in the former that invades and destabilizes the latter (Figure 5c). The binding of the target ligand to the sensing module triggers the displacement of the invading strand and its relocation within the sensing aptamer module, ultimately leading to the restoration of a functional light-up module and fluorescence emission. Using a similar concept, the Mayer lab introduced a strategy in which aptamers, isolated by SELEX [104] or Capture-SELEX [90], are connected to a DFHBI-binding module (Baby-Spinach or Broccoli) via a transducing invader strand that disrupts the folding of the light-up aptamer. Several of these riboswitch-like FRBs have been tested and found to work in living bacteria or mammalian cells to report on metabolite concentrations (see Section 4). Although not demonstrated in living cells, structural reshaping of the light-up aptamer module can also be mediated in trans by the binding of a trigger nucleic acid (e.g., via the formation of a kissing complex) having its annealing capacity under the allosteric control of a small molecule [105].

### 3.3. Mixed Control

Interestingly, both types of control (direct allosteric and strand displacement-mediated) can be elegantly combined by the addition of a ribozyme module [106]. Building on early work by the Breaker lab, which demonstrated the possibility to control self-cleaving ribozymes with small molecules [107], three-party architectures can be designed in which a controlled ribozyme sequesters a key region of the light-up module, maintaining the FRB in a dark state (Figure 5d) [106]. The binding of a target ligand triggers RNA self-cleavage, which, in turn, releases a functional light-up aptamer. Interestingly, the small molecule somehow acts like a catalyst since, upon release from an activated FRB, its interaction with another FRB leads to a new trigger event. This enables a signal amplification that improves the detection limit of the FRB. Even higher sensitivity can be achieved with CHARGE, which combines this strategy with the catalytic hairpin assembly amplification cascade [108]. Despite being highly sensitive, ribozyme-based FRBs suffer from the unidirectionality of their sensing, making them unable to report on the dynamics of the cellular content of the target small molecule.

## 4. Applications of FRBs

Dozens of FRBs (Table 2) have been developed with applications both in living cells and in the extracellular space. Depending on their final use, FRBs can either be directly used or combined with additional modules endowing them with additional properties.

### 4.1. Exploiting RNA Modularity to Extend FRBs Properties

**Figure 6:**
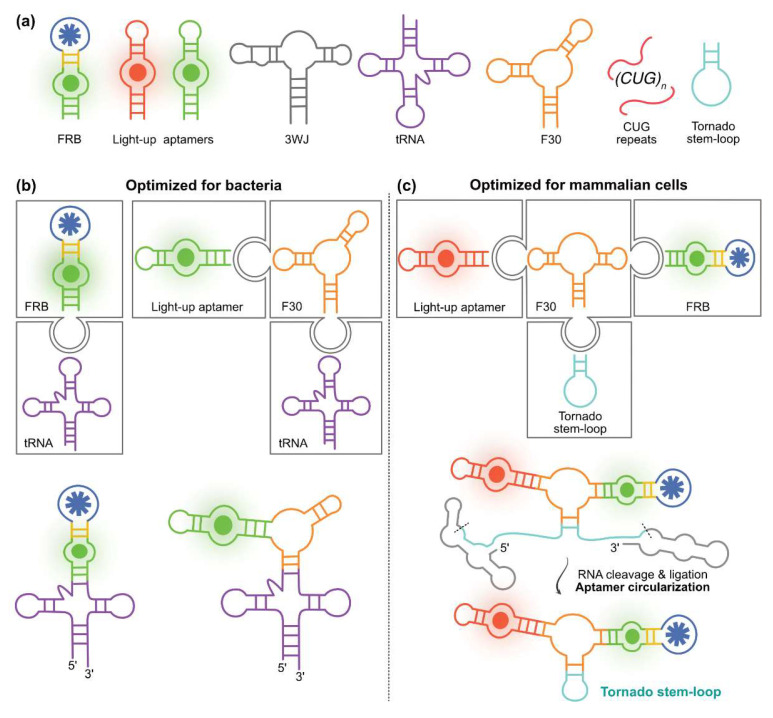
Exploiting RNA modularity. (**a**) Schemes of different additional modules that can be linked to an FRB to increase their accuracy and their stability in living cells. The Tornado stem-loop is shown as expected upon sequence processing. (**b**) FRB-derived constructs optimized biosensing in bacteria and (**c**) in mammalian cells. The modular architecture of the constructs is shown in the upper part. The FRB or the light-up aptamer are connected to other modules via their available stems, depending on the application. The illustration shows only a few possible constructions, highlighting the modularity and variety available for adapting in vivo expression. For example, adding a light-up aptamer allows the construction of a ratiometric biosensor in mammalian cells (right). The Tornado system is represented in a stepwise manner (bottom, right). A linear construct is first generated by transcription. Then, ribozymes surrounding the FRB construct self-cleave and release an anticodon-like structure later recognized by a cellular ligase that catalyzes the circularization of the construct. This circularization event is symbolized by the arrow.

A strong advantage of nucleic acids is their modular organization allowing biosensors to be tailored to specific applications by adding a dedicated module (Figure 6a). For instance, the efficiency of an FRB expressed in living bacteria can be increased by inserting the construct into the anticodon loop of a tRNA-derived scaffold [133]. Not only can the strong folding capacity of the tRNA module assist that of the inserted FRB but the robust tRNA moiety also shields the construct from bacterial RNases activity. Nevertheless, it was later found that this scaffold only partially preserved light-up aptamers and FRBs integrity in bacteria and that better protection can be obtained with the F29 motif (taken form Phi29 phage genomic RNA) or its mutant derivative F30, the latter being particularly well suited for expression in mammalian cells [134]. While efficient in protecting RNAs expressed in bacteria, the effect of these scaffolds was more limited in mammalian systems where a strong exonuclease activity challenges the constructs. Longer term stability can, however, be achieved by adding the so-called Tornado module, which circularizes the construct by exploiting the tandem activity of two ribozymes surrounding the construct and generating an anticodon-like stem-loop that is closed by the ubiquitous endogenous RtcB ligase (Figure 6c) [135]. Finally, although no use of FRB has yet been described in plant cells, their future development would likely benefit from the use of 3WJ, an engineered version of F30 showing superior scaffolding capacity in plant cells [136]. RNA stability can also be enhanced in living cells by appending specific trinucleotide repeats to induce the formation of RNA condensates through phase separation [137]. Accordingly, the addition of a module made of 31 to 121 CUG repeats to light-up aptamers and FRBs drives the formation of fluorogenic aptamer-based RNA condensates (FLARE and FLARE sensors) in living cells (bacteria and mammalian cells) displaying superior resistance to nuclease, thermostability, photostability, and brightness [138].

Thanks to their three-way junction organizations, the F30 and 3WJ motifs can also be used as an assembly platform to which up to three modules can be connected while remaining structurally insulated (Figure 6b,c). This makes possible the design of ratiometric biosensors containing an internal reference, i.e., an FRB fused to a light-up aptamer emitting fluorescence at a different wavelength, as pioneered by the You lab [139]. Fusing red-emitting DNB-based FRBs (sensing tetracycline or c-di-GMP) to the green-emitting light-up aptamer Broccoli through an F30 scaffold yielded FRBs displaying superior accuracy when expressed in living bacteria. Indeed, these self-calibrating biosensors are not affected by the variability in RNA concentration and cellular distribution that may occur within the cell and between cells. Interestingly, a recent report from the Hammond lab suggests that inserting such constructs within a tRNA scaffold (Figure 6b, construct shown on the left part) could even further increase their stability in various bacterial types [140]. Ratiometric FRBs were also produced and validated in mammalian cells by fusing either a Squash-based FRB or a Pepper-based FRB in the F30 scaffold, respectively, with Broccoli or RhoBAST light-up aptamers. The addition of the Tornado components to these constructs (Figure 6c, construct shown on the right part) enabled their stabilization in living cells and allowed exquisite monitoring of target metabolites in living cells.

RNA (or DNA) origami represents another appealing way of developing FRBs. Indeed, this nanotechnology allows nucleic acid modules to be precisely arranged in space and applied to a wide range of functions [141]. For instance, the Apta-FRET sensor uses donor–acceptor systems based on Mango and Spinach aptamers whose relative orientation and distance can be modulated upon recognition of a target small molecule [131]. Inserting an SAM-sensing aptamer in this construct converted it into a FRET-based SAM reporter of superior accuracy, as FRET probes are also inherently self-calibrating. The use of resonance energy transfer was recently extended to bioluminescence by exchanging the donor light-up aptamer for NanoLuc luciferase [142]. As before, these genetically encodable BRET sensors are self-calibrating, with the additional advantage of internally producing exciting photons through luciferase activity. Moreover, placing the light-up module under the allosteric control of an SAM or of a ppGpp-specific sensing aptamer yielded the corresponding FRB.

### 4.2. Intracellular In Vivo FRB Applications

Most FRBs have been developed to sense natural small molecules (Table 2) and monitor the effect of mutations or drug treatment on their intracellular concentration, often with single-cell resolution. The choice of the target was mainly driven by the availability of sensing aptamers either taken from natural riboswitches (e.g., TPP, SAM, ppGpp, and c-di-GMP) or less frequently made of a synthetic one (e.g., tetracycline, theophylline, and 1,6-fructose bisphosphate). Whereas many studies consisted of proof-of-concept experiments (i.e., demonstrating the functionality of the FRB in the complex cellular environment), some reports went a step further and shed more light on FRB potential. Among them, cyclic dinucleotide-specific and SAM-specific FRBs were likely the most exploited (Table 2).

Cyclic dinucleotides are intracellular signaling molecules ubiquitous in all domains of life. Produced by bacteria in response to environmental changes, their synthesis may lead to gene expression reprogramming both in Gram-positive and Gram-negative bacteria [116]. These metabolites are involved in stress responses (especially surface sensing) and biofilm production. Therefore, monitoring their concentration in various cell types and conditions relevant to microbiota (i.e., in aerobic and anaerobic conditions) is important to properly understand biological mechanisms at work [118]. In this context, FRBs are well suited since natural riboswitches responding to different cyclic dinucleotide molecules have been identified and light-up aptamers are not sensitive to oxygen level (conversely, to fluorescent proteins that require oxygen to maturate their fluorophore). The Hammond lab has been particularly active in this field [143] by developing several cyclic dinucleotide-specific FRBs allowing sensing of these metabolites in vitro but also in vivo by live-cell imaging [116] and flow cytometry [118]. After introducing c-di-AMP [115] and c-di-GMP-specific FRBs [114,118], the same group prepared an FRB containing the sensing aptamer of a bioinformatically predicted GEMM-I riboswitch and identified 3′-3′-cGAMP as its natural ligand [113,144]. They also rationally reprogrammed the specificity of this FRB towards 2′-3′-cGAMP [83]. These FRBs were next used to identify Hypr GGDEF and the phosphodiesterase HD GYP as the enzymes, respectively, responsible for 3′,3′-cGAMP synthesis [144] and degradation [145]. In addition to its use in bacteria, the 2′-3′-cGAMP-specific FRB was also applied in mammalian cell lysates to characterize the STING (interferon response stimulator) pathway initiated by the cyclic GMP-AMP synthase (cGAS) activity upon binding dsDNA present in the cytosol [83].

**Figure 7:**
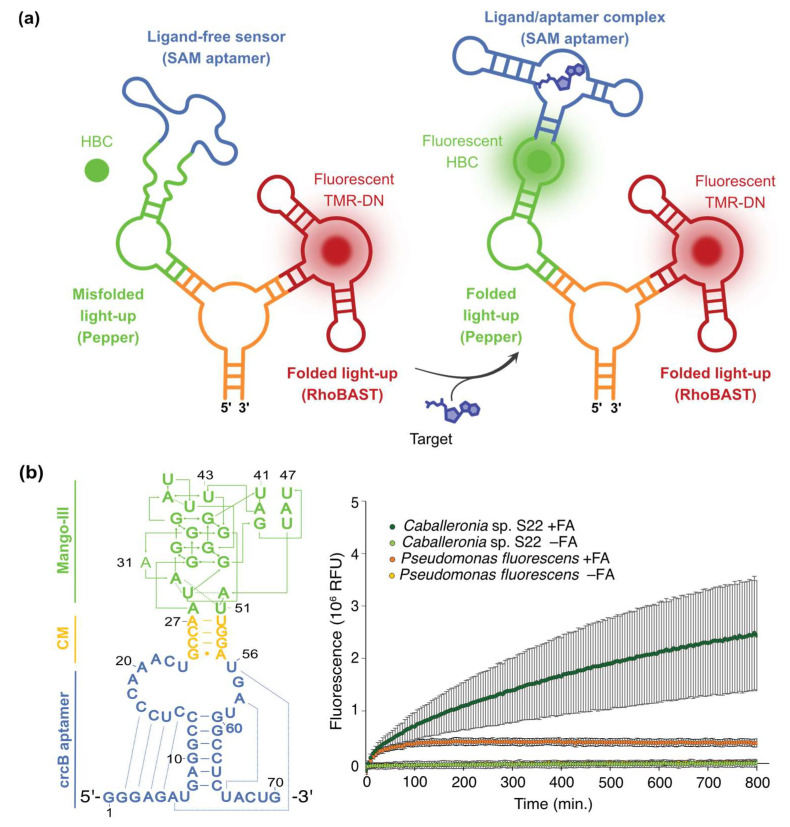
Examples of FRBs applications. (**a**) SAM-Pepper/RhoBAST ratiometric sensor (adapted from [76]). The SAM sensor (blue) exerts an allosteric control on the folding of the light-up Pepper (green). The FRB is inserted into an F30 scaffold (orange) together with the light-up RhoBAST (red). In the absence of SAM, the SAM-Pepper aptamer remains unfolded and dark. Meanwhile, RhoBAST is properly folded and able to activate the fluorescence of TMR-DN (RhoBAST’s cognate fluorogen). In the presence of SAM, the SAM-Pepper aptamer is folded and Pepper activates HBC fluorescence. Correcting the Pepper/HBC fluorescence by that of RhoBAST/TMR-DN allows for normalizing SAM-related fluorescence by the amount of produced biosensor, therefore reducing the impact of cell-to-cell expression variability. The recognition event between the target molecule and the RNA is symbolized by the arrow. (**b**) FluorMango: a fluoride-specific FRB (adapted from [101]). The secondary structure of the biosensor is shown (left), with the sensing aptamer module from crcB riboswitch (in blue) fused to Mango-III light-up aptamer (in green) via a communication module (in orange) identified upon µIVC-seq screening. The addition of the FRB to a mixture containing fluoroacetate as fluorinated substrate and various bacterial strains enables monitoring defluorination activity in real time (right). A defluorinating strain (i.e., *Caballeronia* sp. S22) can easily be discriminated from a non-defluorinating one (i.e., *Pseudomonas fluorescens*). Note that a small background is caused by the free fluoride brought by the fluoroacetate substrate.

S-adenosylmethionine (SAM) is an essential metabolite acting as a universal methyl donor group. As such, it is the cofactor of methyl transferases, a class of enzymes central to epigenetic regulation and associated disorders since they catalyze the methylation of DNA [146], RNA [147], and proteins [148], especially histones. Therefore, monitoring SAM metabolism is of prime importance to properly understand epigenetics and assist the development of drugs targeting related pathways. The design of SAM-specific FRB was greatly eased by the identification of six classes of SAM-specific riboswitches throughout the living kingdoms, the crystal structure of a representative of each class being available [78,149]. Consequently, many SAM-specific FRBs have been developed, most of which are intensiometric (Table 2). Yet, most of these sensors were made of a light-up module (e.g., Broccoli and Corn) comprising a G-quartet core, the stability of which has been questioned in mammalian cells [150]. Although this first generation of SAM-specific FRBs provided a rough evaluation of SAM levels in bacterial and mammalian cells, it could monitor the fluctuation of SAM concentrations in response to a treatment by cycloleucine (an inhibitor of SAM biosynthesis). A significant gain in measurement accuracy was then achieved with the development of ratiometric SAM sensors. Indeed, the use of a SAM-Squash/Broccoli ratiometric sensor allowed precise monitoring of the impact of SAM biosynthesis inhibition but also the impact of culturing conditions on this metabolism with a resolution good enough to discriminate different types of cell response within the same population [66]. Similarly, a SAM-Pepper/RhoBAST ratiometric sensor, an FRB only made of G4-free aptamers (Figure 7a), could be used not only to precisely evaluate the effect of both cycloleucine and SAHA (a drug stimulating SAM synthesis) with single-cell resolution but also to precisely determine the IC50 value of a new drug inhibiting SAM biosynthesis [76]. Such molecular imaging devices could therefore be used in future screening campaigns aimed at discovering new compounds that modulate cell content in SAM to identify drugs for the treatment of epigenetic-related diseases. Moreover, these FRBs could also find application in diagnostics and disease progression monitoring (see below).

### 4.3. Using FRB as Extracellular In Vitro Reporters

Whereas most FRBs have been used to monitor intracellular concentrations of small molecules, there are also many other applications for these molecules in the extracellular space. The ease of producing FRBs in large scale by in vitro transcription stimulated the set-up of several in vitro FBR-based assays to specifically detect target small molecules.

By coupling an S-adenosyl homocysteine (SAH)-specific sensing aptamer to a circularly permuted version of the light-up aptamer Spinach2, the Hammond lab devised a fluorescent high-throughput compatible assay for enzyme activities involved in SAH metabolism [126]. This FRB enabled, for instance, the characterization of inhibitors of methyl transferases. This strategy therefore holds great promise for the discovery of new drugs targeting SAM/SAH metabolism, especially for therapies targeting epigenetic-related diseases. Moreover, together with the SAM-specific FRBs described above, this SAH-specific FRB could find application in diagnostics and disease progression monitoring, as variations in the concentration of both metabolites in patient fluids and tissues have been associated with a wide range of pathologies [151].

FRB were also developed and used to sense metabolites secreted by yeast [123]. In RNA aptamer in droplets (RAPID) technology, libraries of mutant yeast are encapsulated in microfluidic droplets together with a Spinach-derived FRB specific to the target secreted molecule (i.e., tyrosine, tryptophan, or phenylalanine) and DFHBI-1T. Upon incubation, droplets containing the best secreting cells turn the most fluorescent and are specifically sorted from the bulk. Following a similar concept, we recently developed FluorMango (Figure 7b), the first fluoride-specific FRB [101,152]. To do so, we inserted the sequence of the light-up aptamer Mango-III in the P2 helix of the sensor aptamer domain of the crcB riboswitch from *Thermotoga petrophila*. We then used our µIVC-seq screening pipeline (Figure 2c) to identify a handful of communication modules, one of which conferred an impressive turn-on efficiency to FluorMango. The encapsulation of bacteria with the fluorinated substrate, FluorMango, and the fluorogen TO1-Biotin then allowed the detection of defluorinase activity from live bacteria with single-cell resolution.

These different studies illustrate the broad application scope of FRB for the detection of small molecules. Whereas incremental technical progress has improved the performance and stability of FRB in living cells, there is still room for improvement for extracellular applications, especially in complex media.

## 5. Exploring Alternative Chemistries

The great ligand selectivity of FRBs makes them highly valuable tools for small molecule detection out of the cell, with potential applications in diagnostics, microorganism screening, and enzyme and drug discovery. Yet, exploiting the full potential of these biosensors requires them to resist adverse conditions met in the medium. An important limitation of FRBs comes from the inherent instability of their RNA backbone prone to 2′OH-mediated cleavage mainly through nuclease activities. Biosensors can be protected by confining them within lipid particles permeable to the target small molecule but preventing nuclease entry and unwanted release of the biosensors from the vesicles [153]. Although, so far, not tested with FRBs, this strategy may be restricted to the detection of target molecules that are small and hydrophobic enough to cross the membrane. Nuclease activity can also be inactivated through protease treatment. For instance, detection of bacterial fluoride release requires FluorMango-based assay to be performed in the presence of proteinase K [101]. RNA can also be stabilized by adapting the composition of the reaction mixture. Ionic liquid like choline dihydrogen phosphate was shown to preserve malachite-green-specific aptamer light-up function while protecting it from nuclease-mediated degradation. In addition, other organic solvents were shown to protect RNA from degradation [154,155]. Yet, these different treatments may likely interfere with the assay or downstream steps, making more appealing the development of FRBs made of nuclease-resistant chemistry.

The use of DNA-based biosensors represents a first attractive solution, as these molecules are cheap to produce in large quantities by solid-phase chemical synthesis, they are less susceptible to enzymatic and hydrolysis degradation than RNA, and SELEX procedures are well established to identify DNA aptamers. Whereas a handful of light-up DNA aptamers have been described (Table 3), only a very few were further engineered. To our knowledge, the dapoxyl-activating aptamer DAP-10-42 is the only light-up DNA [156] that was engineered into a fluorogenic serotonin-specific biosensor (SERblas) and used as a point-of-care device to detect serotonin in complex media [157]. The light-up aptamer Lettuce was selected for its capacity to activate DFHBI-1T fluorescence emission through what could be the same mechanism as its RNA counterparts Spinach and Broccoli [158]. Although not converted into a small molecule sensor, a split version of Lettuce was engineered to report on the presence of SARS-CoV2 RNA. However, despite their specificity and increased stability, DNA aptamers hardly match RNA aptamers in terms of structural complexity and flexibility [159], which are crucial for the development of specific and dynamic biosensing of small molecules. Moreover, the lack of natural DNA riboswitch further restricts the source of possible target-specific sensing modules and scaffolds.

A large set of synthetic nucleotide chemistries were developed for therapeutic nucleic acids, but they remain poorly explored in the field of fluorogenic biosensing [160,161,162]. For instance, RNA aptamers can be stabilized by modifying the 2′OH group (e.g., 2′fluoro or 2′-O-methyl groups) of their sugar moiety and converting them into xenobiotic nucleic acids (XNA). This allowed, for instance, the preparation of 2′F-Spinach [163] and 2′F-Broccoli [164] aptamers in which all pyrimidines were exchanged for their 2′-fluoro counterparts, resulting in increased stability to degradation. Yet, as this could be expected, introducing such modifications affected aptamers’ properties. While it was beneficial for the capacity of 2′F-Spinach to report on the presence of Pb^2+^ [163] (the G-quartet of Spinach has been shown to sense Pb^2+^ by itself [165]), the same modification introduced in Broccoli led to decreased affinity of the aptamer for K^+^, enabling a potassium sensor to be developed [164]. Resistance to nuclease can also be obtained using enantiomers, i.e., L-RNAs (Spiegelmers) that cannot be processed by natural nucleases [166], as demonstrated by the conversion of Mango-III aptamer into its L counterpart [167]. The light-up aptamer Spinach was also converted into an L-Spinach-based FRB for guanine detection based on the design of the guanine-Spinach FRB developed in 2012 by the Jaffrey Lab as a proof of concept [168]. Even more impressive modifications, consisting of synthetic nucleobases collectively known as Hachimoji, can be specifically introduced into light-up aptamers [169] and used to improve the folding performance of FRBs [170].

However, not all nucleotide modifications are well tolerated by light-up aptamers and FRBs. For instance, the Jaffrey lab showed that modification of nucleobases can significantly drop the function of Broccoli light-up aptamer [171]. Although this adverse effect was used to design a sensor of demethylase activity, most of the time, the introduction of such modifications compromises the function of light-up aptamers and FRBs. Yet, the possibility of enzymatically incorporating these modifications during aptamer synthesis [161] enables in vitro selection and molecular evolution of modified nucleic acids to be undertaken [172,173] and possibly to be applied to the isolation of new generations of light-up aptamers and FRBs with advanced properties.

**Table 3:** Light-up aptamers and FRBs of small molecules made of alternative chemistries.

Aptamer	Chemistry	Fluorogen	Evaluated as FRB of Small Molecule	Target Small Molecule	Other Target	Ref.
AO-binding	DNA	Auramine O	Yes	Serotonin		[157]
AptII-Mini 3-4	DNA	Hoechst	No			[174]
BBR4S3	DNA	Berberine	No			[175]
CV-30S	DNA	Crystal Violet	No			[176]
DAP-10-42	DNA	Dapoxyl	Yes	ATP		[156,177]
DIR 2-1	DNA	DIR	No			[178]
Guanidine	RNA/Hachimoji	DFHBI-T	Yes	Guanidine		[170]
L-Mango-III	L-RNA	TO1-Biotin	No		MicroRNA-155	[167]
L-Spinach	L-RNA	DFHBI	Yes	Guanine		[168]
Lettuce	DNA	DFHBI-T	No		SARS-CoV2 RNA	[158]
Spinach-Hachimoji	DNA/Hachimoji	DFHBI-T	No			[169]
2′F-Broccoli	RNA/2′ Fluoropyrimidine	DFHBI-T	Yes	K^+^		[164]
2′F-Spinach	RNA/2′ Fluoropyrimidine	DFHBI-T	Yes	Pb^2+^		[163]

## 6. Conclusions

This article aimed to review the main strategies used to develop fluorogenic RNA-based biosensors (FRBs) by exploiting the great diversity of natural and synthetic aptamers available. The performance of light-up aptamers and their fluorogens has constantly improved over the years and delivered a new generation of reporting systems (i.e., Pepper, RhoBAST, Squash, and Okra) endowed with all the key requirements expected for efficient reporters: (i) high brightness, (ii) high affinity, (iii) absence of G-quartet, (iv) good folding efficiency, and (v) good cell permeability of a nontoxic fluorogen.

Most FRBs used so far exploited natural riboswitch-derived aptamers, since they evolved to display both the expected structure-switching capacity and high ligand selectivity. Only a small fraction of the 55 known riboswitch families currently known have been used so far. Theoretical calculations based on bioinformatics suggest that nearly 20% of the total riboswitch diversity is still left to be discovered [78].

In addition to this natural reservoir of sensing aptamers, the latest developments have identified general scaffolds like three-way junction motifs to be better suited for specificity reprogramming than more simple, diverse but less constrained libraries. Indeed, imposing a structural context prone to structure switching efficiently restricts the sequence space to the region driving the specificity.

Optimal communication modules functionally linking sensing and reporting modules can now be identified using approaches able to evaluate large sequence libraries (>65,000 sequence permutations). Among them, we believe that the µIVC-seq pipeline represents an important advance [179]. Equally important is the modular nature of nucleic acid that supports the design of FRBs and allows additional functionalities (e.g., nuclease resistance in cells, controlled aggregation, and accurate ratiometric quantification) to the constructs, enabling the development of sensitive and accurate genetically encoded biosensors of small molecules.

The first generation of FRB was mainly restricted to in vitro applications and expression in bacteria in which RNA degradation may be significantly inhibited by inserting the construct into a tRNA scaffold. As described in this review, these first constructs opened new perspectives in drug discovery by enabling the design of enzymatic activity (e.g., demethylase and methyl transferase) or riboswitching (e.g., TPP-responsive riboswitch) reporters suited to the search for specific modulators (i.e., inhibitors, agonists, or antagonists) by high-throughput screening of chemical libraries. Moreover, expressing FRBs in bacteria also shed new light on bacteria biology, as exemplified above by the sensing of cyclic dinucleotides [143]. Indeed, the use of FRB enabled a new riboswitch and several enzymes involved in cyclic dinucleotide metabolism to be identified, each representing a potential target for an antibacterial compound, since cyclic dinucleotides are key mediators of bacterial stress adaptation [180]. Sensing these metabolites in eukaryotic cells would also likely help in addressing complex questions such as the evolution of antiviral immunity [181]. More generally, FRB-mediated sensing of small molecules in mammalian cells may also have a deep impact on the understanding on cell metabolism and assist in the discovery of new drugs, as illustrated by the case of SAM-specific FRBs introduced above.

Although, to our knowledge, no FBR as defined in this review has yet been exploited in a multicellular organism, the functionality of many FRBs in cultured cells together with that established for light-up aptamers like Mango in *C. elegans* [49] and Broccoli in plants [136] supports the feasibility of such application. In such a case, the design of FRBs activating near infrared-emitting fluorogen (e.g., TO3-Biotin, BC, SiR-PEG3-NH2, or DIR-Pro) will likely be instrumental to enable both the efficient excitation of the fluorogen at tissue-penetrating wavelengths and the collection of emitted fluorescence (while limiting the adverse effect of the natural green background autofluorescence of living systems).

Another important future development axis of FRBs will concern their use for extracellular applications, especially in vitro diagnostics, drug screening, and enzyme discovery. For these different applications, the fluorogenic nature of FRBs and their derivative is an important feature by limiting the appearance of false positive signals. Indeed, most DNA probes currently used in fluorescent assays exploit the quenching of fluorescent dyes [182]. Therefore, the possible degradation of the probe by an adverse nuclease activity would lead to the release of the fluorophore and a false positive signal. Conversely, the uncontrolled degradation of the fluorogenic probe keeps it dark, with no signal emitted.

Whereas the first generation of RNA-based biosensors now established the advantages of sensing small molecules using nucleic-acid-based biosensors in terms of substrate selectivity, development time, and production cost, we anticipate that important advances could arise from the use of alternative nucleotide chemistries. Although only a few developments have been attempted in this direction, it makes no doubt that the adaptation of FRBs to alternative chemistries will endow them with exceptional stability, for instance, in human fluids (e.g., urine, blood, and saliva) and extended capacity to detect new targets currently difficult to sense. For instance, the introduction of hydrophobic modifications on nucleobases (like those found on SOMAmers and that enabled the development of aptamers specific to nearly any protein [183]) may increase the potential of FRBs in the detection of hydrophobic compounds. Moreover, adapting FRBs and their derivatives to work in nonconventional solvents may also open up new analytical strategies that are likely to be explored in the near future.

## Figures and Tables

**Table 1:** Principal light-up RNA aptamers and their cognate fluorogen.

Aptamers			Fluorogen		Complex				Ref.
Name	G4	#P ^a^	Name	Ex/Em	*K_D_*	Turn-On	Brightness ^c^	PDB	D/S ^d^
BeCA	no	2	BC-6	478/686	230 nM	51	n.d.	n.d.	[35]
Beetroot	yes	1/2 ^b^	DFAME	514/619	460 nM	40	3825	8EYU	[36,37]
Broccoli	yes	2	DFHBI-1T	470/505	305 nM	800	27,800	n.d.	[38]
			BI	470/505	51 nM	1100	22,500	n.d.	[39]
Chili	yes	2	DMHBI+	413/542	63 nM	n.d.	8400	7OAW	[40,41]
			DMHBO+	456/592	12 nM	n.d.	2100	7OAX	
Corn	yes	1/2 ^b^	DFHO	505/545	70 nM	417	7250	5BJO	[42,43]
DIR-2	no	4	DIR-Pro	600/658	252 nM	20	54,000	6DB8	[44,45]
DNB	n.d.	2	TMR-DN	555/582	350 nM	73	42,430	n.d.	[46]
			SR-DN	572/591	800 nM	56	49,240	n.d.	
iSpinach	yes	2	DFHBI	442/503	920 nM	n.d.	n.d.	5OB3	[47,48]
Mango	yes	1	TO1-B	510/535	4 nM	1300	11,000	5V3F	[49,50]
Mango-II	yes	1	TO1-B	510/535	0.7 nM	1650	17,000	6C63	[51,52]
Mango-III	yes	1	TO1-B	510/535	5.6 nM	4400	43,000	6E8S	[51,53]
MGA	no	2	MG	350/630	117 nM	2360	28,000	1F1T	[54,55]
o-Coral	no	5	Gemini-561	580/596	73 nM	13	82,000	n.d.	[56]
Okra	n.d.	2	ACE	468/505	1.2 nM	664	22,320	n.d.	[57]
Peach	yes	1	TO3-B	637/658	19.6 nM	50	17,800	n.d.	[58]
Pepper	no	2	HBC530	485/530	3.5 nM	3595	43,098	7EOK	[28,59]
			HBC620	577/620	6.1 nM	12,600			
RhoBAST	no	4	TMR-DN	564/590	15.1 nM	26	55,000	8JY0	[60,61]
Riboglow	no	4	Cbl-5xPEG-Atto590	594/622	34 nM	5	37,200	n.d.	[26]
			Cbl-Cy5	646/662	3 nM	2.7	65,750		
SiRA	no	3	SiR-PEG3-NH2	649/662	430 nM	7	84,280	n.d.	[62]
Spinach	yes	2	DFHBI	452/496	562 nM	2000	17,500	4KZD	[63,64]
Spinach2	yes	2	DFHBI	454/498	430 nM	770	29,100	n.d.	[65]
Squash	no	3	DFHO	505/545	53 nM	550	14,760	7KVU	[66,67]

^a^ #P: number of paired regions involved in the structural stabilization of the fluorogen-binding site. ^b^ Works as a homodimer. ^c^ Brightness was calculated by multiplying the absorption coefficient of the complex by its quantum yield. ^d^ The first reference discloses (D) the system, while the others introduce its 3D structure (S).

**Table 2:** FRB of small molecules and their applications.

Target	Light-Up 1 (Fluorogen)	Light-Up 2 (Fluorogen) ^a^	Sensing Aptamer Type	Functional Connection Type	Connection Design	Applications	Ref.
Adenine	Spinach (DFHBI)		Purine riboswitch	Strand displacement	Trial-Error	Proof-of-concept	[102]
	DNB (SR-DN)	Broccoli (DFHBI-1T)	Purine riboswitch	Allosteric	Trial-Error	Live-cell ratiometric imaging	[109]
	Spinach (DFHBI)		Synthetic	Allosteric	Trial-Error	Proof-of-concept	[110]
Adenosine 5-diphosphate (ADP)	Spinach (DFHBI)		Synthetic	Allosteric	Trial-Error	Live-cell imaging	[110]
	BFR (Hoechst 1C)		Synthetic	Strand displacement	Trial-Error	Proof-of-concept	[111]
Adenosine triphosphate (ATP)	MGA (MG)		Synthetic	Allosteric	Trial-Error	Proof-of-concept	[112]
Ag^2+^	Pepper (HBC530)		CC mismatch	Allosteric	Rational design	Live-cell entry imaging	[76]
	Broccoli (DFHBI-1T)		CC mismatch	Allosteric	Rational design	Live-cell entry imaging	[77]
Cyclic AMP (cAMP)	Broccoli (DFHBI-1T)		Synthetic	Ribozyme	Trial-Error	Proof-of-concept	[106]
Cyclic AMP-GMP (3′-5′ cGAMP)	Spinach (DFHBI)		GEMM-Ib	Allosteric	Trial-Error	Live-cell imaging and riboswitch discovery	[113]
Cyclic AMP-GMP (c-AMP-GMP)	Spinach (DFHBI)		G20A GEMM-I	Allosteric	Trial-Error	Live-cell imaging and in vitro enzymatic assay	[114]
Cyclic di-AMP (cdiA)	Spinach (DFHBI-1T)		yuaA	Allosteric	Trial-Error	Live-cell imaging	[115]
	Spinach2 (DFHBI)		yuaA	Allosteric	Trial-Error	Live-cell imaging and cytometry profiling	[116]
Cyclic di-GMP (c-di-GMP)	Spinach (DFHBI)		Vc2	Allosteric	Trial-Error	Proof-of-concept	[117]
	Spinach (DFHBI)		GEMM-I	Allosteric	Trial-Error	Live-cell imaging	[114]
	DNB (SR-DN)	Broccoli (DFHBI-1T)	GEMM-I	Allosteric	Trial-Error	Live-cell ratiometric imaging	[109]
	Spinach2 (DFHBI/DFHBI-1T)		GEMM-I	Allosteric	Trial-Error	Live-cell imaging under aerobic and anaerobic conditions	[118]
	Broccoli (DFHBI-1T)		GEMM-I	Ribozyme	Trial-Error	Proof-of-concept	[106]
FMN	MGA (MG)		Synthetic	Allosteric	Trial-Error	Proof-of-concept	[112]
Glycine	Spinach (DFHBI-1T)		Tandem glycine aptamer (*B. subtillis*)	Strand displacement	Screening	Thermodynamic characterization of a riboswitch	[119]
Guanine	Spinach (DFHBI)		Synthetic	Allosteric	Trial-Error	Proof-of-concept	[110]
	Spinach (DFHBI)		Purine riboswitch	Strand Displacement	Trial-Error	Live-cell imaging	[102]
	Broccoli (DFHBI-1T)		Purine riboswitch	Allosteric	Trial-Error	Proof-of-concept	[120]
	Pepper (HBC)		Purine riboswitch	Allosteric	Trial-Error	Live-cell entry imaging	[76]
Guanosine-5′-triphosphate (GTP)	Spinach (DFHBI)		Synthetic	Allosteric	Trial-Error	Proof-of-concept	[110]
L-Dopa	Broccoli (DFHBI)		Synthetic	Allosteric	Screening	Proof-of-concept	[121]
L-Dopa/Dopamine	Broccoli (DFHBI-1T)		Synthetic	Allosteric	Trial-Error	Proof-of-concept post SELEX screening of aptamer	[84]
Nickel (Ni^2+^), Cobalt (Co^2+^), Iron (Fe^2+^)	Spinach2 (DFHBI-1T)		NiCo, czcD	Allosteric	Trial-Error	Determine riboswitch ligand specificity	[122]
Phenylalanine	Spinach2 (DFHBI or DFHBI-1T)		Synthetic	Allosteric	Trial-Error	Screening of secreting microbes	[123]
ppGpp	Broccoli (DFHBI-1T)		(p)ppGpp riboswitch	Allosteric	Trial-Error	Live-cell imaging	[124]
	Broccoli (DFHBI-1T)		(p)ppGpp riboswitch	Allosteric	Trial-Error	Paper-based biosensing	[125]
S-adenosyl-homocysteine (SAH)	Spinach2 (DFHBI)		SAH riboswitch	Allosteric	Trial-Error	Live-cell imaging/In vitro methyltransferase assay	[126]
S-adenosylmethionine (SAM)	Spinach (DFHBI)		SAM-I riboswitch	Strand displacement	Trial-Error	Live-cell imaging	[102]
	Spinach (DFHBI)		Synthetic	Allosteric	Trial-Error	Live-cell imaging	[110]
	Corn (DFHO)		SAM-III circular permuted	Allosteric	Trial-Error	Live-cell imaging and drug effect monitoring	[127]
	Red Broccoli (OBI)		SAM riboswitch	Allosteric	Trial-Error	Live-cell imaging	[128]
	Pepper (HBC530 or HBC620)		SAM-III riboswitch	Allosteric	Trial-Error	Live-cell imaging and drug effect monitoring	[129]
	Spinach (DFHBI-1T)		SAM-III riboswitch	Allosteric	Screening	Proof-of-concept	[72]
	Cladogenetic B2 (Isa-5a) Cladogenetic G2 (DFHBI-1T)		SAM-III riboswitch	Allosteric	Screening from [72]	Live-cell imaging	[130]
	Squash (DFHO)	Broccoli (BI)	SAM-III riboswitch	Allosteric	Selection	Accurate detection and drug effect monitoring	[66]
	Pepper (HBC530)	RhoBAST (TMR-DN)	SAM-III riboswitch	Allosteric	Trial-Error	Accurate detection and drug effect monitoring	[76]
	Corn (DFHO)		SAM-III riboswitch	Allosteric	Trial-Error	Live-cell imaging and drug effect monitoring	[120]
	Spinach (DFHBI)		SAM-I riboswitch	Strand Displacement	Trial-Error	Live-cell imaging	[102]
	Spinach (DFHBI-1T)	Mango (YO-3)	SAM-III riboswitch	FRET	Rational design	Proof-of-concept	[131]
	Spinach (DFHBI-1T)		SAM-III riboswitch	Allosteric	Screening	Accurate detection and drug effect monitoring	[72]
Tetracycline	DNB (SR-DN)	Broccoli (DFHBI-1T)	Synthetic	Allosteric	Trial-Error	Live-cell ratiometric imaging	[109]
	Pepper (HBC530)		Synthetic	Allosteric	Trial-Error	Live-cell entry imaging	[76]
	Brocolli (DFHBI-1T)		Synthetic	Allosteric	Trial-Error	Paper-based biosensing	[125]
Theophylline	MGA (MG)		Synthetic	Allosteric	Trial-Error	Proof-of-concept	[112]
	iSpinach (DFHBI-1T)		Synthetic	Allosteric	Screening	Proof-of-concept	[100]
	Cladogenetic B2 (Isa-5a) Cladogenetic G2 (DFHBI-1T)		Synthetic	Allosteric	Screening from [72]	Live-cell imaging	[130]
	Broccoli (DFHBI-1T)		Synthetic	Ribozyme	Trial-Error	Live-cell imaging and cytometry profiling	[106]
	MGA (MG)		Synthetic	Kissing	Rational design	Proof-of-concept	[105]
TPP	Spinach (DFHBI)		TPP riboswitch	Strand Displacement	Trial-Error	Live-cell imaging and drug screening	[102]
	Broccoli (DFHBI-1T)		TPP riboswitch	Ribozyme	Trial-Error	Proof-of-concept	[106]
	Broccoli (DFHBI-1T) Red Broccoli (OBI)		Synthetic	Strand displacement	Selection	Proof-of-concept	[90]
Tryptophane	Spinach2 (DFHBI or DFHBI-1T)		Synthetic	Allosteric	Trial-Error	Screening of secreting microbes	[123]
Tyrosine	Broccoli or Spinach2 (DFHBI-1T)		Synthetic	Allosteric	Trial-Error	Proof-of-concept	[132]
	Spinach2 (DFHBI or DFHBI-1T)		Synthetic	Allosteric	Trial-Error	Screening of secreting microbes	[123]
2′,3′-cGAMP	Spinach2 (DFHBI-1T)		G103A GEMM-II	Allosteric	Trial-Error	Live-cell imaging and characterization of cGAS-cGAMP-STING pathway	[83]
5-HTP	Broccolli (DFHBI-1T)		Synthetic	Strand displacement	Trial-Error	Proof-of-concept	[84]
5-HTP/Serotonine	Broccoli (DFHBI-1T)		Synthetic	Allosteric	Trial-Error	Live-cell imaging	[120]

^a^ When a ratiometric sensor was used.

## Abbreviations

2′FY	2′ fluorinated pyrimidine
3WJ	3-way junction
5-HT	5-hydroxytryptamine
5-HTP	5-hydroxytryptophan
ADP	Adenosine diphosphate
AMP	Adenosine monophosphate
AO	Auramine O
apta-FRET	aptamer-based Förster resonance energy transfer
ATP	Adenosine triphosphate
BC	Benzopyrylium Coumarin
BFR	Blue fluorescent RNA
BRET	Bioluminescence Resonance Energy Transfer
c-di-GMP	cyclic-dimeric guanosine monophosphate
c-AMP	cyclic adenosine monophosphate
Cbl	Cobalamin
c-diA	cyclic di-AMP
cGAMP	cyclic GMP-AMP
cGAS	cyclic GMP-AMP synthase
CHARGE	Catalytic Hairpin Assembly RNA circuit that is Genetically Encoded
CM	Communication Module
CV	Crystal violet
DAP	Dapoxyl DNA aptamer
DFAMF	3,5-difluoro-4-hydroxybenzylidene imidazolinone-2-acrylate methyl
DFHBI	3,5-difluoro-4-hydroxybenzylidene imidazolinone
DFHBI1-T	3,5-difluoro-4-hydroxybenzylidene-1-trifluoroethyl-imidazolinone
DFHO	3,5-difluoro-4-hydroxybenzylidene-imidazolinone-2-oxime
DIR	Dimethylindole red
DMHBI	3,5-dimethoxy-4-hydroxybenzylidene imidazolone
DNA	Deoxyribonucleic acid
DNB	Dinitroaniline-binding aptamer
dsDNA	double stranded DNA
FACS	Fluorescence-activated Cell Sorting
FLARE	Fluorogenic Aptamer-based RNA condensates
FMN	Flavin mononucleotide
FP	Fluorescent protein
FRB	Fluorogenic RNA-based biosensor
FRET	Fluorescence Resonance Energy Transfer
GEMM	Genes for the Environment, for Membranes and for Motility
GFP	Green Fluorescent Protein
GRAP	Gene-linked RNA aptamer particle
GTP	Guanosine 5′-triphosphate
HBC	4-((2-hydroxyethyl)(methyl)amino)-benzylidene)-cyanophenylacetonitrile
IC50	Half-maximal inhibitory concentration
IVT	In vitro transcription
MG	Malachite green
DIR-2	Dimethylindole red 2
PCR	Polymerase chain reaction
ppGpp	Guanosine tetraphosphate
RAPID	RNA Aptamer In Droplets
RhoBAST	Rhodamine Binding Aptamer for Super-resolution Imaging Techniques
RNA	Ribonucleic acid
SAH	S-Adenosyl-L-homocysteine
SAHA	Suberoylanilide hydroxamic acid
SAM	S-adenosylmethionine
SARS-CoV-2	Severe acute respiratory syndrome coronavirus 2
SELEX	Systematic Evolution of Ligands by EXponential enrichment
SerBLAS	Serotonin-responsive binary light-up aptameric sensor
SiRA	Silicon Rhodamine-Binding Aptamer
SR-DN	Sulforhodamine B-dinitroaniline
SRB	Sulforhodamine B
TMR	Tetramethylrhodamine
TMR-DN	Tetramethylrhodamine dinitroaniline
TO	Thiazole orange
TORNADO	Twister-optimized RNA for durable overexpression
TPP	Thiamine pyrophosphate
tRNA	transfert RNA
µIVC	Microfluidic-assisted in vitro compartmentalization
XNA	Xeno nucleic acid
xpt-pbuX	Xanthine phosphoribosyltransferase- xanthine-specific purine permease
